# Loss of antiphospholipid antibody positivity decreases the risk of recurrent thrombosis in thrombotic antiphospholipid syndrome

**DOI:** 10.1093/rheumatology/keaf679

**Published:** 2025-12-14

**Authors:** Pedro Gaspar, Ana Rita Cruz-Machado, Ana Mafalda Abrantes, Filipa Costa, Inês Parreira, Ana Rita Lopes, Ryan Costa-Silva, Ana Teodósio Chícharo, Joana Rosa Martins, João Pedro Marques, Diogo Santos, Vasco C Romão, Luis Graca, João E Fonseca

**Affiliations:** Internal Medicine Department, ULS Santa Maria, Centro Académico de Medicina de Lisboa, Lisbon, Portugal; GIMM – Gulbenkian Institute for Molecular Medicine, Centro Académico de Medicina de Lisboa, Lisbon, Portugal; Faculdade de Medicina, Universidade de Lisboa, Centro Académico de Medicina de Lisboa, Lisbon, Portugal; Faculdade de Medicina, Universidade de Lisboa, Centro Académico de Medicina de Lisboa, Lisbon, Portugal; Rheumatology Department, ULS Santa Maria, Centro Académico de Medicina de Lisboa, Lisbon, Portugal; Internal Medicine Department, ULS Santa Maria, Centro Académico de Medicina de Lisboa, Lisbon, Portugal; Faculdade de Medicina, Universidade de Lisboa, Centro Académico de Medicina de Lisboa, Lisbon, Portugal; iNOVA4Health, Nova Medical School, Lisbon, Portugal; Faculdade de Medicina, Universidade de Lisboa, Centro Académico de Medicina de Lisboa, Lisbon, Portugal; Rheumatology Department, ULS Santa Maria, Centro Académico de Medicina de Lisboa, Lisbon, Portugal; Internal Medicine Department, ULS Santa Maria, Centro Académico de Medicina de Lisboa, Lisbon, Portugal; Faculdade de Medicina, Universidade de Lisboa, Centro Académico de Medicina de Lisboa, Lisbon, Portugal; Rheumatology Department, ULS Santa Maria, Centro Académico de Medicina de Lisboa, Lisbon, Portugal; Internal Medicine Department, ULS Santa Maria, Centro Académico de Medicina de Lisboa, Lisbon, Portugal; Faculdade de Medicina, Universidade de Lisboa, Centro Académico de Medicina de Lisboa, Lisbon, Portugal; Rheumatology Department, ULS Santa Maria, Centro Académico de Medicina de Lisboa, Lisbon, Portugal; Rheumatology Department, ULS do Algarve, Faro, Portugal; Internal Medicine Department, ULS Santa Maria, Centro Académico de Medicina de Lisboa, Lisbon, Portugal; Faculdade de Medicina, Universidade de Lisboa, Centro Académico de Medicina de Lisboa, Lisbon, Portugal; Internal Medicine Department, ULS Santa Maria, Centro Académico de Medicina de Lisboa, Lisbon, Portugal; Faculdade de Medicina, Universidade de Lisboa, Centro Académico de Medicina de Lisboa, Lisbon, Portugal; Faculdade de Medicina, Universidade de Lisboa, Centro Académico de Medicina de Lisboa, Lisbon, Portugal; Rheumatology Department, ULS Santa Maria, Centro Académico de Medicina de Lisboa, Lisbon, Portugal; GIMM – Gulbenkian Institute for Molecular Medicine, Centro Académico de Medicina de Lisboa, Lisbon, Portugal; Faculdade de Medicina, Universidade de Lisboa, Centro Académico de Medicina de Lisboa, Lisbon, Portugal; Faculdade de Medicina, Universidade de Lisboa, Centro Académico de Medicina de Lisboa, Lisbon, Portugal; Rheumatology Department, ULS Santa Maria, Centro Académico de Medicina de Lisboa, Lisbon, Portugal

**Keywords:** antiphospholipid syndrome, antiphospholipid antibodies, prognosis, recurrence, seroconversion, thrombosis

## Abstract

**Objective:**

To establish the predictive value of antiphospholipid antibody (aPL) negativization on recurrent thrombosis in thrombotic antiphospholipid syndrome (APS).

**Methods:**

Retrospective cohort study including all consecutive adult patients with APS followed at Hospital de Santa Maria, Lisbon, Portugal, up to December 2024. At diagnosis, all patients were positive for solid-phase aPL. Then, patients were categorized in two groups according to aPL during follow-up: (i) persistently aPL-positive; (ii) aPL-negativization defined as previous aPL-positive and current aPL-negative (two consecutive times, ≥12 months apart). Outcomes included recurrent thrombosis under antithrombotic therapy. Predictors of aPL-negativization and recurrent thrombosis were assessed using Cox regression analysis.

**Results:**

Of the 116 included patients (female, 78.4%; primary APS, 72.1%; triple positivity, 40.5%), totalling 1084 patient years (PY), 31.9% became aPL-negative (3.4 events/100PY). The follow-up time was similar between aPL-positive and aPL-negativization groups (*P* = 0.681), and before and after aPL-negativization (*P* = 0.072). At diagnosis, triple aPL-positivity predicted against aPL-negativization, while primary APS predicted aPL-negativization. Forty-five patients (38.8%) experienced 68 recurrences (4.2 events/100PY). Age at APS onset, arterial thrombosis and persistent triple aPL-positivity predicted recurrence. Globally, aPL-negativization did not predict recurrence (HR 0.58, 95%CI 0.29–1.14). Among patients who became aPL-negative, the cumulative incidence of recurrent thrombosis did not differ before aPL-negativization (HR 0.52, 95%CI 0.26–1.06) but dropped by 88% after aPL-negativization (HR 0.12, 95%CI 0.05–0.58), even after adjusting for age, primary APS and type of initial thrombosis (*P* = 0.006).

**Conclusion:**

The risk of recurrent thrombosis drops significantly after aPL-negativization, highlighting the utility of serial aPL monitoring in clinical practice.

## Graphical abstract

**Figure:**
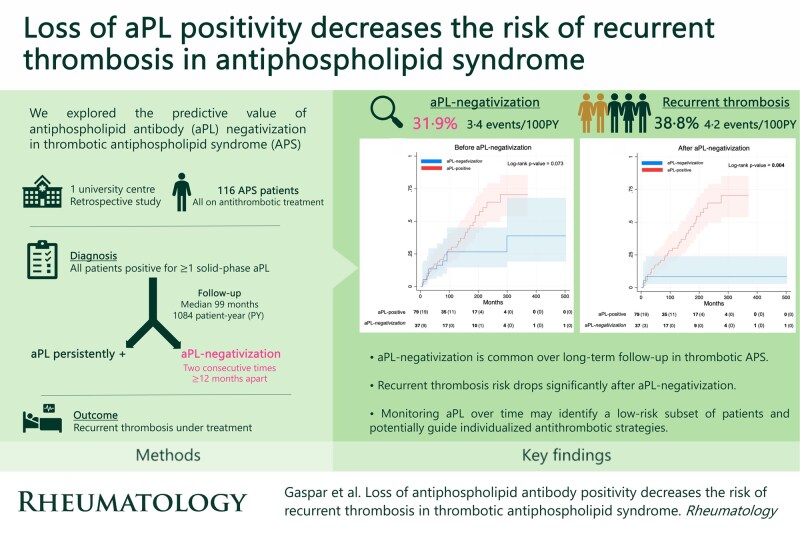

Rheumatology key messages

- Antiphospholipid antibody (aPL) negativization is common over long-term follow-up in thrombotic antiphospholipid syndrome (APS).
- Recurrent thrombosis risk drops significantly after aPL negativization.
- Monitoring aPL over time may identify a group of patients with a more favourable prognosis.

## Introduction

Thrombotic antiphospholipid syndrome (APS) is defined by the presence of venous, arterial or microvascular thrombosis in the setting of persistently positive antiphospholipid antibodies (aPL) [1]. The thrombotic risk in APS depends on the type, number, titre and persistence of aPL [2]. Patients with a high-risk aPL profile (e.g. triple positive) are at greatest risk of recurrent thrombosis [3–5], which contributes to progressive organ damage, morbidity and mortality [6–8].

aPL titres fluctuate over time and may become undetectable [9]. The prevalence of aPL-negativization in 259 APS patients was 8.9% at 5 years [10]. Other studies reported higher rates, ranging from 29% [11] to 59% [12]. Differences in assay type, cut-off values and follow-up duration partly explain this variability, but collectively indicate that aPL levels are dynamic in a significant proportion of patients.

Whether aPL-negativization has prognostic implications remains unclear. Some studies have suggested that anticoagulation may be safely discontinued after aPL-negativization [13], challenging the current recommendation for life-long treatment with vitamin K antagonist (VKA) [2]. Most supporting data derive from small retrospective cohorts reporting few or no thrombotic recurrences after treatment withdrawal [14–17]. Conversely, Medina *et al.* [11] observed that 46% of patients relapsed within 60 months of aPL loss despite ongoing VKA use. These inconsistencies highlight the uncertainty surrounding the prognostic value of aPL-negativization in thrombotic APS.

Given the established pathogenic role of aPL in promoting thrombosis [18], we hypothesized that achieving aPL-negativization would significantly reduce future risk of thrombosis. Therefore, we aimed to investigate the impact of aPL-negativization on the recurrent thrombosis in patients with thrombotic APS.

## Methods

### Study design

This was a retrospective open cohort study including all consecutive patients with thrombotic APS followed at the Internal Medicine and Rheumatology clinics at Hospital de Santa Maria, Unidade Local de Saúde Santa Maria, Centro Académico de Medicina de Lisboa (CAML), Lisbon, Portugal, up to December 2024.

The study was approved by the CAML ethics committee (N° 235/23) and conducted in accordance with the Declaration of Helsinki. Only data obtained during routine clinical care were analysed. No identifiable patient information collected; therefore, written informed consent was waived.

### Participants

Eligible patients fulfilled the entry clinical criteria for APS according to the 2023 ACR/EULAR classification criteria [1] and were persistently positive for at least one aPL, namely, lupus anticoagulant (LA), IgM and/or IgG anti-cardiolipin (aCL) and/or anti-β2-glycoprotein (aβ2GPI) on two consecutive measurements at least 12 weeks apart.

In accordance with current guidelines [1, 2], we report no standardized institutional protocol dictating the timing or rationale for subsequent aPL repeat testing during patient’s follow-up, or to guide treatment adjustments. aPL reassessment was therefore performed at the discretion of the treating physician, and treatment adjustments, when made, were individualized and in accordance with the patient’s preference.

A key aspect of our study was the ability to assess aPL-negativization during the study period, representing our primary exposure measure. To ensure data integrity, all available aPL results were assessed (including before and after aPL-negativization, when applicable), and only patients with aPL retesting after the diagnosis were included. Given the unreliability of LA testing during anticoagulation [19] and its limited interpretability in retrospective analyses, only solid-phase aPL assays were used to define follow-up aPL status. Accordingly, all patients were positive for aCL and/or aβ2GPI at diagnosis, regardless of LA status. Patients were then stratified according to aPL status during follow-up: (i) aPL-positive, defined as persistent positivity (IgM and/or IgG aCL and/or aβ2GPI) until the end of study; and (ii) aPL-negative, representing those who achieved aPL-negativization, defined as two consecutive negative results at least 12 months apart.

Patients with obstetric-only APS, isolated LA positivity, age <18 years, <1 year of follow-up, indeterminate timing of aPL-negativization, or who were asymptomatic aPL carriers were excluded.

### Procedures

Data were obtained through a structured manual review of electronic medical records using predefined criteria (Supplementary Data S1). A comprehensive set of variables was collected for each patient, including: (i) sex (self-reported among the following options: male, female, other); (ii) current age (at December 2024); (iii) age at/year of disease onset; (iv) type of APS and associated autoimmune disease; (v) other medical disorders; (vi) type of presenting thrombosis; (vii) cumulative thrombotic manifestations, including recurrent thrombosis (until December 2024); (viii) treatment; and (ix) aPL profile at diagnosis and during follow-up. Data were collected by P.G., A.R.C.-M., A.M.A., F.C., I.P., A.R.L., R.C.-S., A.T.C., J.R.M., J.P.M. and D.S. between June and November 2024.

Clinical manifestations were obtained from medical records and confirmed by imaging, laboratory and/or histopathological findings when appropriate. The total number of cumulative thrombotic events was determined. Recurrent events were defined as those occurring during antithrombotic therapy and represented our primary outcome measure. During follow-up, aPL testing adhered to the classification criteria applicable at the time. LA was determined according to the International Society on Thrombosis and Haemostasis recommendations [20]. Solid-phase IgM/IgG aCL and aβ2GPI antibodies were determined by enzyme-linked immunosorbent assay (ELISA) (QUANTA Lite^®^ ELISAs, INOVA Diagnostics, San Diego, CA, USA) until 2014, and chemiluminescence immunoassay (CLIA) (QUANTA-Flash^®^, INOVA Diagnostics, San Diego, CA, USA) thereafter. Positivity thresholds followed manufacturer-recommended cut-offs.

### Statistical analysis

Descriptive statistics were used to characterize the study population. Continuous variables are expressed as median (interquartile range) and categorical variables as number (%), and compared using the Mann–Whitney *U* test, and Pearson’s chi-squared test or Fisher’s exact test when adequate. Patients were followed up until the last visit (December 2024), occurrence of the primary outcome, or lost to follow-up. Lost to follow-up patients were censured at the date of last contact. To evaluate the potential impact of assay variability, we compared patients who repeated aPL measurements in the same in-house laboratory using the same assay kits (‘consistent testing’) or externally and/or with different kits (‘inconsistent testing’). To assess potential bias from non-standardized aPL retesting, we compared both the total number of aPL measurements and the frequency of aPL testing per year of follow-up according to aPL-negativization status and recurrent thrombosis. Uni- and multivariate Cox regression models with aPL-negativization and recurrent thrombosis as dependent variables were performed, and Kaplan–Meier failure curves were computed to evaluate the cumulative incidence of recurrent thrombosis based on the aPL-negativization status and timing. To avoid immortal time bias, aPL-negativization was modelled as a time-dependent covariate, and Cox regression was used for inference. A Simon–Makuch failure curve was generated to visualize recurrence according to time-dependent aPL status, and Kaplan–Meier plots were produced descriptively to illustrate cumulative incidence before and after aPL-negativization. Variables were eligible for multivariate analysis considering their clinical/biological significance and/or their statistically significance between-group differences. Only variables that were present in at least 10% of the cohort were included in the model, hence enhancing the robustness of the effect estimates. Multicollinearity of variables was assessed. We handled missing data using a list-wise deletion approach; we dropped from a specific analysis any patient who had a missing value in that specific variable (i.e. number/[total number – dropped values]). This applied only for certain comorbid conditions; there were no missing data for our primary exposure and outcome measures. Additional stratified Cox analyses were performed according to aPL specificity (aCL *vs* aβ2GPI) and isotype (IgM only *vs* any IgG positivity) at diagnosis and at the end of study to assess potential differential effects on aPL-negativization and recurrent thrombosis. Statistical significance was set as a two-sided *P*-value of <0.05. The statistical analysis was performed using STATA^®^ version 16.

## Results

### Clinical characterization and demographics

Of 267 eligible patients, 116 (43.4%) met the inclusion criteria (Supplementary Fig. S1). Demographic, clinical and laboratory characteristics are summarized in Table 1. Most patients were female (78.4%), with a median age of 52 years and a median follow-up time of 99 months, totalling 1084 patient-years (PY). The majority (72.1%) had primary APS. Secondary APS was more frequently associated with systemic lupus erythematosus (75.0%).

**Table 1.** Characteristics of the study population

	Total N = 116
Clinical characteristics	
Female sex	91 (78.4)
Current age (years)	52 (42.5–63)
Age at APS onset (years)	38 (25–49.5)
Time of follow-up (months)	99 (46.5–186.5)
APS characterization	
Primary APS	72 (72.1)
Secondary APS	44 (37.9)
SLE	33 (28.5 | 75.0)^j^
SLE-like^a^	8 (6.9 | 18.2)^j^
Other^b^	3 (2.6 | 6.8)^j^
Type of first thrombotic event	
Arterial	57 (49.1)
Venous	58 (50.0)
Microvascular^c^	1 (0.9)
Obstetric morbidity	22/73 (30.1)
aPL profile	
At diagnosis	
Lupus anticoagulant	75 (64.7)
Anticardiolipin (IgM/IgG)	97 (83.6)
Anti-β2-glycoprotein I (IgM/IgG)	75 (64.7)
Triple positive	47 (40.5)
At end of study	
Lupus anticoagulant	55 (47.4)
Anticardiolipin (IgM/IgG)	66 (56.9)
Anti-β2-glycoprotein I (IgM/IgG)	65 (56.0)
Triple positive	43 (37.1)
aPL-negativization	37 (31.9)
Medical disorders	
At diagnosis	
High blood pressure	30 (25.9)
Hyperlipidaemia	19 (16.4)
Obesity	25 (21.6)
Diabetes	4 (3.5)
Chronic kidney disease	4 (3.5)
At end of study	
High blood pressure	58 (50.0)
Hyperlipidaemia	58 (50.0)
Obesity	30 (25.9)
Diabetes	8 (6.9)
Chronic kidney disease	12 (10.3)
Thrombophilia^d^	6/59 (10.2)
Smoking	57 (49·1)
Treatment (ever-present)	
Anticoagulation	108 (93.1)
Vitamin K antagonist	104 (89.7 | 96.3)^j^
Low molecular weight heparin	26 (22.2 | 24.1)^j^
Direct oral anticoagulants^e^	23 (19.8 | 21.3)^j^
Antiplatelet agents^f^	67 (57.8)
Statin	64 (55.2)
Hydroxychloroquine	50 (43.1)
Steroids	37 (31.9)
DMARDs	25 (21.6)
Azathioprine	19 (16.4 | 76.0)^j^
Methotrexate	6 (5.2 | 24.0)^j^
Mycophenolate mofetil	7 (6.0 | 28.0)^j^
Other^g^	5 (4.3 | 20.0)^j^
Biologics	5 (4.3)
Rituximab	4 (3.5 | 80.0)^j^
Other^h^	1 (0.9 | 20.0)^j^
Outcomes	
Number thrombotic events	2 (1–3)
Recurrent thrombosis	45 (38.8)
DIAPS ≥1	89 (76.7)
Early damage^i^	48 (41.4)
DIAPS initial	0 (0–1) 0.5 ± 0.6
DIAPS ≥3	37 (31.9)
DIAPS final	2 (1–3) 1.8 ± 1.5

Data are shown as number (%) and median (interquartile range) when appropriate. DIAPS is also reported as mean ± standard deviation for comparison with similar studies. The denominator is provided if it differs from the group total. See Supplementary Data S1 for further detail on medical disorders’ definitions.

a SLE-like included the following: undifferentiated connective tissue disease (*n* = 7), mixed connective tissue disease (*n* = 1).

b Other included the following: Sjögren syndrome (*n* = 2), inflammatory spondyloarthropathy (*n* = 1).

c These include renal thrombotic microangiopathy and microvascular skin thrombosis.

d Inherited thrombophilia included the following: protein S deficiency (*n* = 4/76, 5.3%), factor V Leiden (*n* = 3/66, 4.5%), 20210a prothrombin mutation (*n* = 2/64, 3.1%) and protein C deficiency (*n* = 2/78, 2.6%).

e DOAC included the following: rivaroxaban (*n* = 15), apixaban (*n* = 7) and dabigatran (*n* = 1).

f Antiplatelets included the following: low-dose aspirin (*n* = 65) and clopidogrel (*n* = 2).

g Other DMARDs included the following: cyclophosphamide (*n* = 5), intravenous immunoglobulin (*n* = 2) and ciclosporin (*n* = 2).

h Other biologics included the following: secukinumab (*n* = 1).

i Early damage refers to damage acquired during the first six months after disease onset.

j Within-subgroup proportions.

aPL: antiphospholipid antibodies; APS: antiphospholipid syndrome; DIAPS: damage index for antiphospholipid syndrome; DMARDs: disease modifying anti-rheumatic drugs.

**Table 2.** Univariate hazard ratio identifying predictors of aPL-negativization and recurrent thrombosis

	aPL-negativization		Recurrent thrombosis	
	HR (95% CI)	*P*-value	HR (95% CI)	*P*-value
Clinical characteristics				
Male sex	1.70 (0.80–3.62)	0.168	1.60 (0.81–3.16)	0.180
Current age (years)^a^	0.99 (0.97–1.02)	0.776	1.02 (0.99–1.05)	0.072
Age at APS onset (years)^a^	1.01 (0.99–1.03)	0.407	1.03 (1.01–1.06)	**0.002**
Time of follow-up (months)^a^	0.99 (0.98–0.99)	**0.001**	0.99 (0.99–1.00)	**0.017**
APS characterization				
Primary APS	2.12 (1.04–4.31)	**0.038**	1.13 (0.62–2.04)	0.694
Secondary APS	0.47 (0.23–0.96)		0.89 (0.49–1.61)	
Type of first thrombotic event				
Arterial	0.99 (0.52–1.88)	0.967	2.16 (1.19–3.94)	**0.012**
Venous	1.04 (0.55–1.99)	0.898	0.47 (0.26–0.86)	**0.014**
Obstetric morbidity	0.51 (0.20–1.29)	0.155	1.56 (0.73–3.35)	0.252
aPL profile				
At diagnosis				
Lupus anticoagulant	0.35 (0.18–0.67)	**0.001**	1.46 (0.75–2.83)	0.262
Anticardiolipin	0·69 (0.30–1.58)	0.386	1·56 (0·61–3·97)	0·346
Anti-β2-glycoprotein I	0.40 (0.21–0.76)	**0.005**	1.17 (0.63–2.19)	0.614
Triple	0.13 (0.05–0.37)	**<0.001**	1.80 (0.99–3.24)	0.050
At end of study				
Lupus anticoagulant	–	–	1.26 (0.70–2.26)	0.447
Anticardiolipin	–	–	2.23 (1.16–4.28)	**0.016**
Anti-β2-glycoprotein I	–	–	1.60 (0.86–2.97)	0.140
Triple	–	–	1.96 (1.09–3.51)	**0.025**
aPL-negativization	–	–	0.58 (0.29–1.14)	0.115
Medical disorders				
At diagnosis				
High blood pressure	1.05 (0.46–2.40)	0.906	1.47 (0.74–2.95)	0.273
Hyperlipidaemia	169 (0.73–3.88)	0.217	2.32 (1.09–4.92)	**0.028**
Obesity	0.50 (0.19–1.27)	0.145	1.07 (0.51–2.22)	0.864
Diabetes	^b^	—	1.69 (0.22–12.69)	0.610
Chronic kidney disease	1.65 (0.22–12.13)	0.625	1.14 (0.15–8.39)	0.899
At end of study				
High blood pressure	0.99 (0.52–1.90)	0.981	0.85 (0.47–1.53)	0.582
Hyperlipidaemia	0.81 (0.42–1.55)	0.517	1.30 (0.72–2.34)	0.383
Obesity	0.45 (0.19–1.07)	0.071	0.77 (0.38–1.55)	0.459
Diabetes	0.35 (0.05–2.57)	0.304	0.96 (0.23–3.96)	0.952
Chronic kidney disease	0.89 (0.27–2.88)	0.840	0.95 (0.34–2.66)	0.918
Thrombophilia	1.33 (0.39–4.55)	0.648	2.68 (0.89–8.06)	0.079
Smoking	1.07 (0.56–2.05)	0.836	1.59 (0.88–2.88)	0.122
Treatment (ever-present)				
Anticoagulation	0.42 (0.16–1.07)	0.070	4.14 (0.57–30.08)	0.161
Vitamin K antagonist	0.58 (0.24–1.40)	0.226	5.91 (0.81–42.93)	0.079
Direct oral anticoagulant	0.82 (0.32–2.12)	0.689	1.23 (0.52–2.43)	0.764
Antiplatelets	0.88 (0.46–1.73)	0.727	1.25 (0.66–2.36)	0.486
Statin	0.83 (0.44–1.59)	0.580	2.16 (1.13–4.12)	**0.019**
Hydroxychloroquine	0.63 (0.33–1.21)	0.164	0.92 (0.51–1.65)	0.770
Steroids	0.46 (0.22–0.98)	**0.043**	1.15 (0.64–2.09)	0.638
DMARDs	0.90 (0.42–1.90)	0.777	0.82 (0.42–1.63)	0.581
Biologics	1.50 (0.36–6.27)	0.576	0.36 (0.05–2.63)	0.314

Data are shown as univariate hazard ratio (HR) and 95% confidence interval (CI). See Supplementary Data S1 for further detail on medical disorders’ definitions.

Significant results are highlighted in bold.

a Per one-unite increase HR.

b None of the patients with diabetes at diagnosis become negative for aPL.

aPL: antiphospholipid antibodies; APS: antiphospholipid syndrome; DMARDs: disease modifying anti-rheumatic drugs.

A total of 246 cumulative thrombotic events were identified (Supplementary Table S1), most commonly venous thromboembolism (51.6%), with microvascular thrombosis observed in only 2.0% of cases. During the follow-up, all patients received antithrombotic therapy: 93.1% were treated with anticoagulation (96.3% on VKA, all warfarin) and 57.8% received antiplatelet agents.

At diagnosis, high blood pressure, obesity and hyperlipidaemia were the most prevalent comorbidities (Table 1). The prevalence of all comorbidities rose during follow-up, but statistical significance was reached only for hypertension and hyperlipidaemia, both among persistently aPL-positive and aPL-negative patients (Supplementary Table S2).

### aPL-negativization

At diagnosis, IgM/IgG aCL, IgM/IgG aβ2GPI, and triple positivity were observed in 83.6%, 64.7% and 40.5% of patients, respectively (Table 1). After a median follow-up of 67 months, 37 (31.9%) patients became negative for all solid-phase aPL (aPL-negativization group; 3.4 events/100 PY). None of these patients reverted to aPL-positive during subsequent follow-up.

aPL testing practices are detailed in Supplementary Tables S3 and S4. Although patients who achieved aPL-negativization underwent slightly more total aPL measurements than those who remained positive (median 9 *vs* 8 tests, *P* = 0.048), the annual testing frequency was comparable between groups (median 1.4 *vs* 1.2 tests/year, *P* = 0.536) (Supplementary Table S3). Overall, 90.5% of patients had aPL tested at our centre. This included longitudinal monitoring of 97.5% of patients who remained persistently aPL-positive, and confirmation of aPL-negativization in 75.7% of those who became negative. A change in assay methodology occurred in 26.7% of repeated in-house determinations, reflecting the institutional transition from ELISA to CLIA in 2015 (see Methods). Among patients who became aPL-negative, 43.2% had negativization confirmed with an alternative assay, while 29.1% of persistently positive patients had repeat results reconfirmed by a different assay (Supplementary Table S4). Testing consistency did not differ significantly between patients who achieved aPL-negativization and those who remained positive (56.8% *vs* 70.9%, *P* = 0.133) (Supplementary Table S3).

No significant clinical and demographic differences were observed between patients who become aPL-negative and those who remained aPL-positive (Supplementary Table S5). Specifically, the median follow-up was similar between groups (88, 62–149 *vs* 111, 38–192 months, *P* = 0.681), as were the observation periods before and after aPL-negativization among those who became aPL-negative (28, 13–48 *vs* 32, 17–94 months, *P* = 0.076).

Predictors of aPL-negativization are detailed in Table 2. Primary APS was associated with higher likelihood of aPL-negativization, while LA positivity, aβ2GPI (IgM/IgG) and triple positivity at diagnosis predicted against it. Stratification by aPL specificity and isotype (Supplementary Table S6) showed that single-positive profiles (aCL-only or aβ2GPI-only) were associated with significantly higher rates of aPL-negativization regardless of aPL isotype (only IgM *vs* IgM/IgG). Conversely, double-positive profiles (aCL and aβ2GPI) were less likely to negativize despite considering only IgM subtype. No other demographic, comorbidity or treatment predicted aPL-negativization.

Treatment practices regarding the maintenance, discontinuation or initiation of anticoagulation in our cohort are summarized in Supplementary Table S7. Nine patients discontinued anticoagulation during follow-up: six after aPL-negativization and three despite persistent positivity. Among the six who became negative, two had never received antiplatelet therapy and remained untreated, two continued antiplatelet therapy and two initiated low-dose aspirin after anticoagulation withdrawal. All three persistently positive patients who discontinued anticoagulation remained on antiplatelet therapy. No recurrent thrombosis occurred in either group during a median follow-up of 99 (46–180) months.

### Thrombosis recurrence

Forty-five patients (38.8%) experienced 68 recurrent thrombotic events (4.2 events/100 PY), with 42.2% reporting ≥2 events (maximum four in two patients). Supplementary Tables S8 and S9 summarize the clinical characteristics and event details for recurrent thrombosis among patients who became aPL-negative (events: *n* = 17; patients: *n* = 11) and those who remain aPL-positive (events: *n* = 51; patients: *n* = 34).

Patients who developed recurrent thrombosis underwent fewer aPL measurements per year than those without recurrence (median 1.0 *vs* 1.5 tests/year, *P* = 0.003), despite similar total number of aPL determinations performed (Supplementary Table S3).

In univariate Cox regression analysis (Table 2), older age at APS onset and arterial presenting thrombosis predicted recurrence. Primary APS and baseline aPL profile were not predictive of recurrent thrombosis, whereas persistent aCL positivity and triple positivity were predictors. In stratified analyses based on aPL specificity and isotype at diagnosis (Supplementary Table S6), no antibody subgroups showed a significant association with recurrent thrombosis. Among comorbidities, only hyperlipidaemia at diagnosis predicted recurrence.

**Figure 1.**
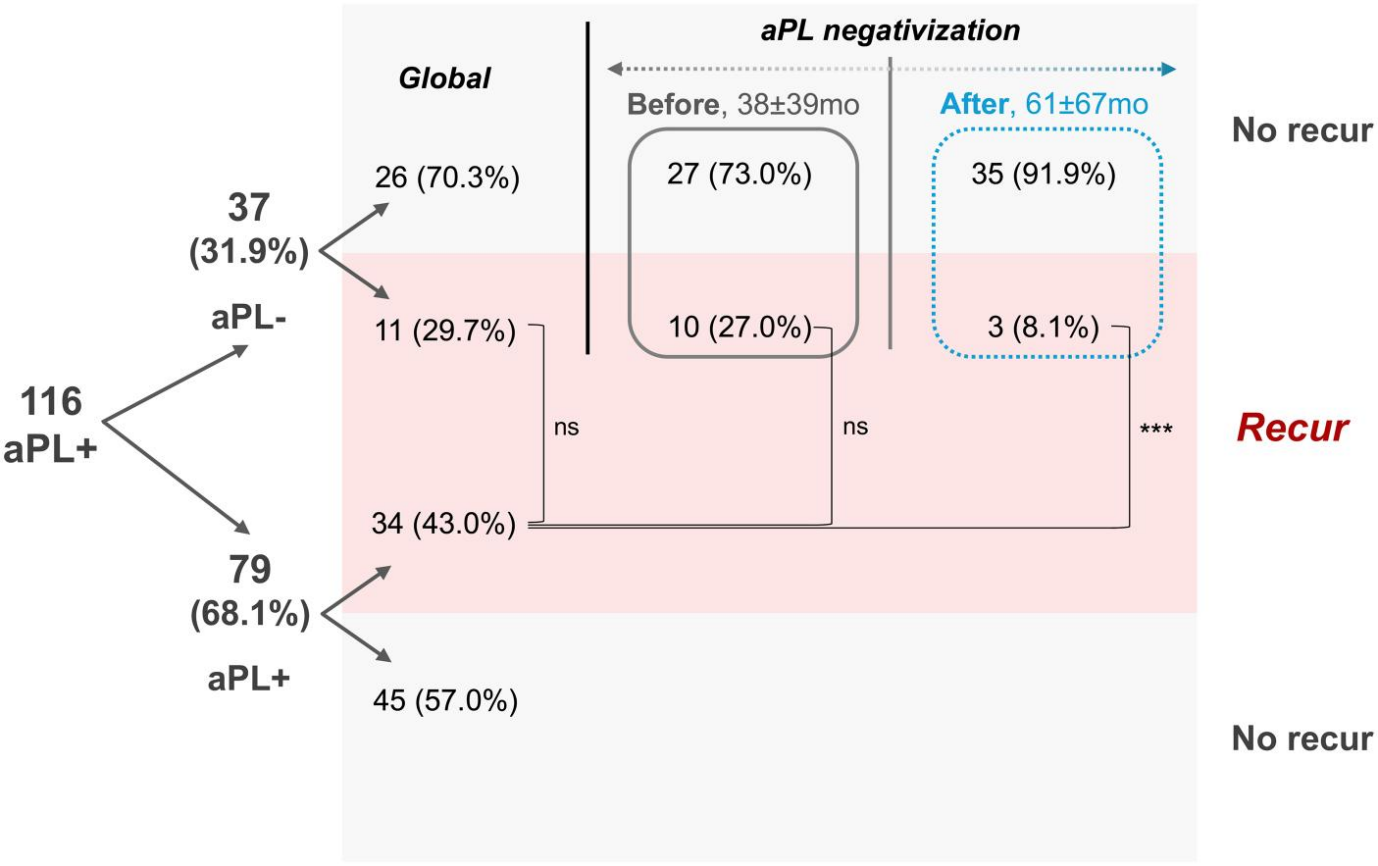
Schematic view of the prevalence of recurrent thrombosis organized by aPL-negativization status and timing. Among patients who become aPL-negative (31.9%), the prevalence of recurrent thrombosis was further analysed according to the temporal relationship with aPL-negativization (before: grey *vs* after: blue). Two patients experienced one thrombotic recurrence before and after aPL-negativization. Proportions were compared using Test of Proportions. ***P <0.001. aPL: antiphospholipid antibodies; mo: months; ns: non-significant.

Fig. 1 illustrates the prevalence of recurrent thrombosis according to aPL status and timing of negativization. Globally, the prevalence of recurrent thrombosis did not differ between patients who became aPL-negative and those who remained positive (29.7% *vs* 43.0%, *P* = 0.170). Then we analysed the timing of recurrence in respect to aPL-negativization occurrence. Compared with patients who remain positive for aPL, patients who became aPL-negative had a similar proportion of recurrent thrombosis before aPL-negativization (43.0% *vs* 27.0%, *P* = 0.098), and a significant decrease in the proportion of recurrent thrombosis after aPL-negativization (43.0% *vs* 8.1%, *P* <0.001).

**Figure 2.**
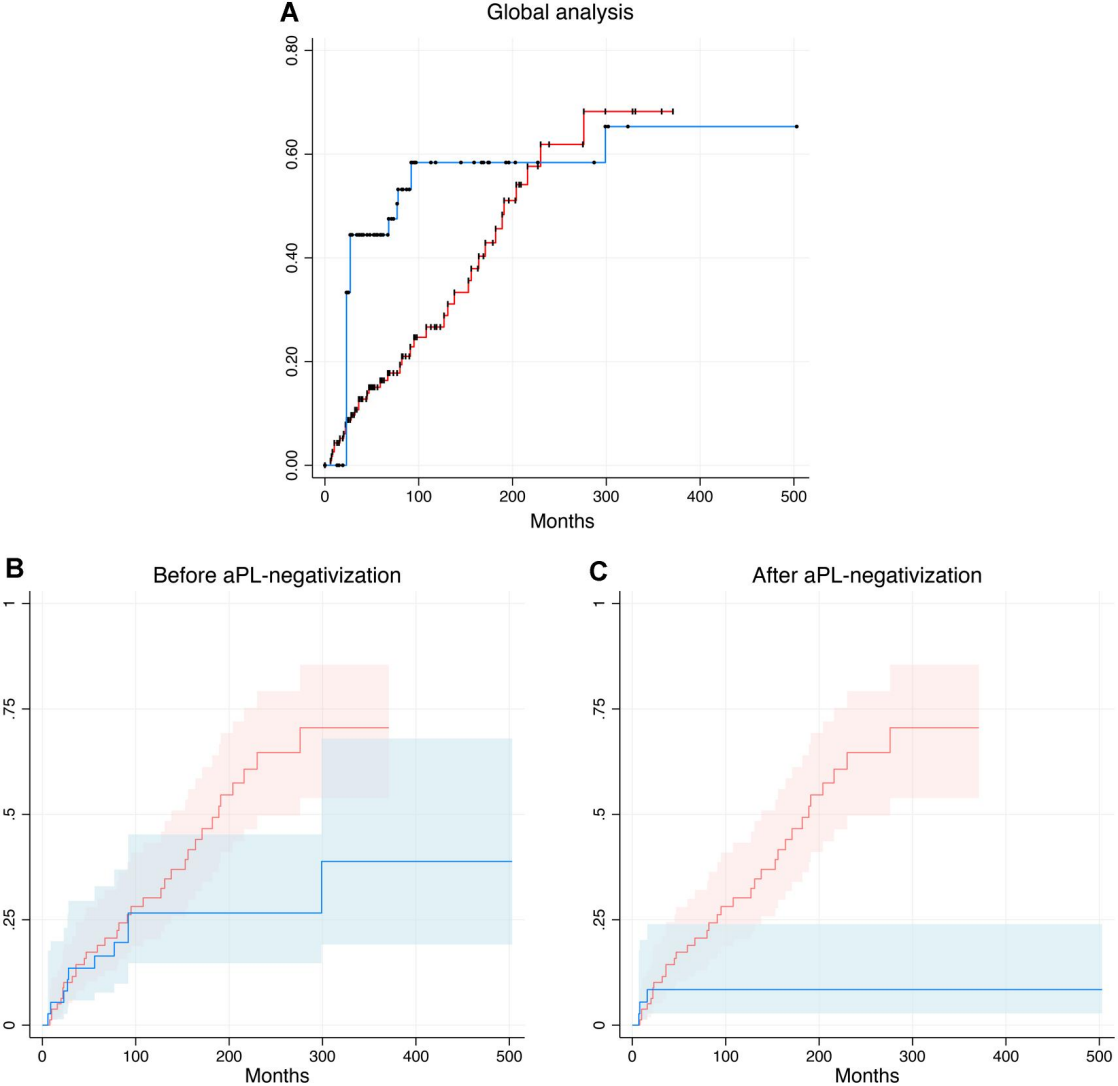
Recurrent thrombosis according to aPL status during follow-up. (**A**) A Simon–Makuch time-dependent failure curve, in which patients contribute person-time to the aPL-positive state until the date of aPL-negativization and to the aPL-negative state thereafter. Event times are marked along each curve as circles (aPL-negativization curve) and pipes (aPL-positive curve). (**B**) and (**C**) show Kaplan–Meier failure curves comparing cumulative incidence of recurrent thrombosis before (**B**) and after (**C**) aPL-negativization, respectively, among patients who became aPL-negative (in blue) *vs* those who remained persistently aPL-positive (in red). Numbers below the curves represent patients at risk (number of events). Curves are provided for descriptive purposes; statistical inference is based on the time-dependent univariate Cox regression model

The Simon–Makuch plot (Fig. 2A) illustrates this dynamic according to time-dependent aPL status: all patients contributed risk time to the aPL-positive group until the date of aPL-negativization, and to the aPL-negative group thereafter if event-free. The cumulative incidence of recurrent thrombosis continued to rise among patients who remained aPL-positive, whereas it plateaued after aPL-negativization. Kaplan–Meier analysis stratified by timing (Fig. 2B and C) confirmed comparable recurrent thrombosis risk before negativization (log-rank *P* = 0.073) and a significantly lower risk afterwards (log-rank *P* = 0.004). Consistently, in the time-dependent Cox model, the hazard of recurrence did not differ before aPL-negativization (HR 0.52, 95% CI 0.26–1.06) but decreased by 88% after aPL-negativization (HR 0.12, 95% CI 0.05–0.58). This association remained significant after adjustment for age at APS onset, type of index thrombosis and APS category (HR 0.19, 95% CI 0.06–0.63; *P* = 0.006) (Supplementary Table S10).

## Discussion

This study provides a detailed characterization of the predictors of aPL-negativization and recurrent thrombosis, and the long-term prognostic value of aPL-negativization. We demonstrate, for the first time, that aPL-negativization is independently associated with a significant reduction in the hazard of recurrent thrombosis among patients adhering to antithrombotic treatment. These findings suggest that aPL-negativization may represent a turning point in the natural history of APS and support the concept that aPL actively contribute to thrombotic risk.

In thrombotic APS, diagnosis and classification heavily rely on the presence, type and titres of aPL [1, 21]. However, no clinical guidelines currently recommend serial aPL monitoring in established disease [1, 2], and there is no standardized definition of clinically relevant changes in aPL levels, particularly for aPL-negativization [9]. In our study, aPL-negativization was deemed as the persistent absence of all criteria solid-phase aPL in at least two consecutive determinations ≥12 months apart. A key finding in our cohort was the sustained aPL-negative status among all patients who achieved aPL-negativization, reinforcing the robustness of our definition and its clinical relevance. This consistent negativization throughout the remainder of the follow-up period enabled us to exclude patients with fluctuating profiles, which can occur around thrombotic events [22].

Our study expands on previous literature by offering a comprehensive assessment of aPL dynamics and thrombotic outcomes in a well-characterized cohort. The observed aPL-negativization rate (∼32%) aligns with prior reports [10–12, 14, 22–24], reinforcing the notion that aPL fluctuate meaningfully over time. Importantly, patients who achieved aPL-negativization had a thrombotic risk comparable to persistently aPL-positive individuals before negativization but showed a significant risk reduction afterward – an effect independent of treatment exposure, comorbidities, follow-up duration, baseline phenotype or aPL specificity and isotype. These findings highlight the potential value of longitudinal aPL to refine long-term risk stratification, particularly when considering long-term management strategies.

Persistent positivity for all three criteria aPL is a well-established marker of high thrombotic risk [2, 5]. This profile not only predisposes patients with established thrombotic APS to recurrent thromboembolic events [3–5] but also increases the likelihood of a first thrombosis among asymptomatic aPL carriers [25]. Our findings corroborate this, showing an approximately two-fold increase hazard of recurrent thrombosis in persistently triple positive patients. Additionally, traditional cardiovascular risk factors remain important contributors to thrombosis in thrombotic APS [4, 5]. In our cohort, hyperlipidaemia at diagnosis more than doubled the hazard of recurrent thrombosis, underscoring the need for aggressive management of coexisting risk factors [2], particularly in those with arterial thrombosis, which emerged as a strong predictor of further events.

Current guidelines recommend lifelong anticoagulation for patients with thrombotic APS, irrespective of aPL dynamics [2]. Consistent with recent observations [26], our findings suggest that aPL-negativization may identify a subset of patients with a more favourable thrombotic risk profile. Although treatment de-escalating has been discussed in carefully selected cases [12–17], only a few patients in our cohort discontinued anticoagulation after aPL-negativization. Even though no recurrent thrombosis occurred, this number is too small to draw conclusions supporting anticoagulation tapering or withdrawal in clinical practice. These results are therefore hypothesis-generating rather than practice-changing. Any treatment de-escalation strategies must be tested in prospective controlled studies before being considered clinically.

These findings raise new questions about the determinants and mechanisms underlying aPL-negativization. We identified primary APS as a predictor of aPL-negativization. Conversely, LA positivity, aβ2GPI and triple positivity at diagnosis predicted against it. This may partly reflect differences in aPL profiles between primary and secondary APS (that is, patients with primary APS are predominantly represented in the aCL-positive group). While a comprehensive comparison between primary and secondary APS was beyond the scope of this work, this observation warrants validation in future research.

There is also a need to better understand the immunological mechanisms underlying aPL-negativization and its clinical relevance. Although aPL are implicated in thromboinflammatory pathways [18, 27–29] our findings are observational and do not allow mechanistic inference. It remains unknown whether, when and to what extent these aPL-mediated prothrombotic effects are reversible following aPL-negativization. Future studies integrating longitudinal aPL profiling with functional assays are required to clarify whether aPL-negativization reflects a true immunological shift or simply laboratory variability. Understanding these processes could ultimately help identify immunological targets beyond anticoagulation in APS.

These findings must be interpreted considering several important limitations. First, this was a retrospective study conducted at a single centre, which may limit generalizability. Second, the absence of an institutional protocol for the timing and frequency of aPL testing during follow-up, with the decision based on the attending physician’s judgment, introduces potential variability. Importantly, our rigorous inclusion criteria, similar follow-up duration across groups, and uniform definition of aPL-negativization help to reduce the impact of these limitations. Moreover, the frequency of aPL monitoring per year was similar between patients who did and did not seroconvert, and patients who experienced recurrent thrombosis were in fact tested less frequently. These findings reduce the likelihood that our results were driven by surveillance bias. Third, although most aPL measurements were performed in a single laboratory, changes in assay methods and some external testing could have introduced variability. However, our sensitivity analyses showed that testing consistency was similar across groups. Moreover, misclassification bias is unlikely given the higher analytical sensitivity of the CLIA (our institution’s standard since 2015) used during most of the follow-up period [21, 30]. This suggests that aPL-negativization was accurately assigned. Fourth, isolated LA-positive patients were excluded due to the unreliability of LA testing during anticoagulation, a methodological decision that avoided incorrect exposure classification but may have excluded a relevant high-risk subgroup [2]. Fifth, although our definition of aPL-negativization was consistent with prior studies [9], there is no universally accepted standard. In particular, follow-up time varied across patients, and long-term estimates should be interpreted with caution, as only a minority of patients (18.9%) remained under observation beyond 200 months. Finally, few patients discontinued anticoagulation, limiting our ability to make definitive statements about the safety of withdrawal based solely on aPL status. Future multicentre prospective studies are warranted to confirm our findings and to establish standardized strategies for longitudinal aPL assessment.

Notwithstanding these limitations, our findings have important implications. From a clinical perspective, repeated aPL testing, though not foreseen in the clinical management of these patients [2], should be considered beyond initial diagnosis, as it could help identify a subset of patients with a more favourable thrombotic risk trajectory. From a research perspective, prospective multicentre studies with standardized longitudinal aPL monitoring are warranted to validate aPL-negativization as a prognostic biomarker and to evaluate whether it may contribute to individualized anticoagulation strategies.

To conclude, our study demonstrates that solid-phase aPL-negativization occurred in nearly one-third of patients with thrombotic APS and was independently associated with a significantly lower risk of recurrent thrombosis after seroconversion. While less frequent in individuals with high-risk aPL profiles, aPL-negativization identifies a subgroup of patients with a more favourable long-term thrombotic outcome. These results support the concept that serological evolution carries prognostic relevance in APS and justify future investigation into its potential role in treatment stratification.

## Supplementary Material

keaf679_Supplementary_Data

## Data Availability

The data underlying this article are available in the article and in its online supplementary material.
