# Supplementary material for: Loss of antiphospholipid antibody positivity decreases the risk of recurrent thrombosis in thrombotic antiphospholipid syndrome
Source: Rheumatology (Oxford). 2025 Dec 14;65(1):keaf679. doi: 10.1093/rheumatology/keaf679 (PMC12790813; doi:10.1093/rheumatology/keaf679)
Supplement: keaf679_Supplementary_Data [file keaf679_supplementary_data.docx]

***Loss of antiphospholipid antibody positivity decreases the risk of recurrent thrombosis in thrombotic antiphospholipid syndrome***

Pedro Gaspar et al.

**Supplementary material**

| **Content** | **Legend** | **Page** |
| --- | --- | --- |
| **Supplementary Figure S1** | Flow chart showing patients selection process. | 2 |
| **Supplementary Data S1** | Definitions. | 3 |
| **Supplementary Table S1** | Distribution of thrombotic manifestations. | 5 |
| **Supplementary Table S2** | Comparison of the prevalence of aPL profile positivity and medical disorders at diagnosis and at the end of the study in all patients. | 6 |
| **Supplementary Table S3** | Descriptive analysis on the aPL testing practices during the study period. | 7 |
| **Supplementary Table S4** | Frequency of aPL testing and testing consistency according to aPL-negativization status and recurrent thrombosis. | 7 |
| **Supplementary Table S5** | Comparison of demographic and clinical characteristics between patients with and without aPL-negativization and recurrent thrombosis. | 8 |
| **Supplementary Table S6** | Stratified Cox regression analysis by aPL specificity and isotype. | 10 |
| **Supplementary Table S7** | Management practices regarding the maintenance, discontinuation or initiation of anticoagulation. | 11 |
| **Supplementary Table S8** | Characterization of recurrent thrombosis in patients who become aPL-negative. | 12 |
| **Supplementary Table S9** | Characterization of recurrent thrombosis in patients who remained aPL-positive. | 13 |
| **Supplementary Table S10** | Multivariate hazard ratio identifying predictors of recurrent thrombosis. | 15 |

**Supplementary Figure S1**. Patient’s selection process. ^a^ aPL-*negativization* impossible to be determined included those patients with no / inconsistent serial aPL monitoring during follow-up. aPL, antiphospholipid antibodies, APS, antiphospholipid syndrome.

**Supplementary Data S1. Definitions.**

| Variable | Definition |
| --- | --- |
| APS onset | Taken as the time the first clinical event related to APS occurred. |
| APS diagnosis | Taken as the time the patient had two consecutive positive aPL determinations at least 12 weeks apart, within five years of the last thrombotic event. |
| Current age | Age, in years, at the end of the study (December 2024) / lost to follow-up / death. |
| Age at onset | Age, in years, at the time the first clinical event related to APS occurred. |
| Time of follow-up | Taken as time, in months, from APS diagnosis until the end of study (December 2024) / lost to follow-up / death. |
| Type of presenting thrombosis | Taken as arterial, venous or microvascular thrombosis at the time of APS onset. |
| Obstetric APS | Taken as any obstetric morbidity fulfilling the clinical criteria of the Sydney classification criteria. |
| Recurrent thrombosis | Thrombotic event (arterial, venous, or microvascular) occurring during treatment with antithrombotic therapy during the follow-up period. |
| High blood pressure | Taken as ever present. Stated as (at least one of the following in two consecutive appointments): 1) systolic ≥140mmHg or diastolic ≥90mmHg; 2) and/or medical record of the diagnosis regardless of its cause or severity. |
| Diabetes mellitus | Diabetes mellitus type 1 or 2 taken as ever present. Stated as (at least one of the following): 1) fasting glucose level ≥126mg/dL (if known); 2) random glucose level ≥200mg/dL (if known); 3) Medical record of the diagnosis regardless of its cause and severity. |
| Obesity | Taken as ever present. Stated as (at least one of the following): body mass index ≥ 30 Kg/m^2^ and/or medical record of the diagnosis. |
| Hyperlipidaemia | Taken as ever present. Stated as (at least one of the following): 1) total cholesterol ≥190mg/dL (if known) or LDL-cholesterol above normal range; 2) Medical record of the diagnosis. |
| Chronic kidney disease | Taken as ever present. Stated as (at least one of the following): 1) estimated glomerular filtration rate <60mL/min; 2) Medical record of the diagnosis. |
| Inherited thrombophilia | Taken as ever present. Include any of the following: 1) Factor V Leiden; 2) protein C deficiency; 3) protein S deficiency; 4) antithrombin deficiency; 5) 20210a prothrombin mutation. |
| Smoking | Taken as ever present. Included both current or previous smoking regardless of the total amount of daily cigarettes. |
| SLE | Classified according to the SLICC classification criteria.^1^ |
| UCTD | Classified according to international classification criteria.^2^ |
| Sjögren Syndrome | Classified according to the 2016 ACR-EULAR classification criteria for primary Sjögren’s Syndrome.^3^ |
| Lupus anticoagulant | According to ISTH recommendations.^4^ |
| aCL (IgM and/or IgG) | Determined by enzyme-linked immunosorbent assay (QUANTA Lite® ELISAs, INOVA Diagnostics, San Diego, CA, USA) until 2014, and chemiluminescence immunoassay (QUANTA-Flash®, INOVA Diagnostics, San Diego, CA, USA) after 2015. Threshold values to define positivity were based on manufacturer’s cutoff. |
| aβ2GPI (IgM and/or IgG) | Determined by enzyme-linked immunosorbent assay (QUANTA Lite® ELISAs, INOVA Diagnostics, San Diego, CA, USA) until 2014, and chemiluminescence immunoassay (QUANTA-Flash®, INOVA Diagnostics, San Diego, CA, USA) after 2015. Threshold values to define positivity were based on manufacturer’s cutoff. |
| Anticoagulation | Taken as ever present (at least six months). Includes any kind of anticoagulants. |
| Antiplatelet therapy | Taken as ever present (at least six months). Includes any antiplatelet agent. |
| Statin | Taken as ever present (at least six months). Includes any statin at any dose. |
| Hydroxychloroquine | Taken as ever present (at least six months). Any dose. |
| Steroids | Taken as ever present (at least six months). Includes only equivalent dose of prednisolone ≥7.5mg/day. |
| DMARDs | Taken as ever present (at least six months). Includes any kind of disease modifying anti-rheumatic drugs. |
| Biologics | Taken as ever present. Includes any kind of biologic drugs ever given. |
| Damage | Defined according to the DIAPS.^5^ |
| Early damage | DIAPS ≥1 at the first six months after disease onset. |
| Any damage | DIAPS ≥1. |
| Severe damage | DIAPS ≥3. |

aβ2GPI, anti-β2 glycoprotein I; aCL, anticardiolipin; aPL, antiphospholipid antibodies; APS, antiphospholipid syndrome; CRNM, clinically relevant non-major bleeding; DIAPS, damage index for antiphospholipid syndrome; DMARDs, disease modifying anti-rheumatic drugs; ISTH, International society for Thrombosis and Haemostasis; SLE, systemic lupus erythematosus; UCTD, undifferentiated connective tissue disease.

**References:**

1 Petri M, Orbai A-M, Alarcón GS, *et al.* Derivation and validation of the Systemic Lupus International Collaborating Clinics classification criteria for systemic lupus erythematosus. *Arthritis Rheum* 2012; **64**: 2677–86.

2 Mosca M, Tani C, Vagnani S, Carli L, Bombardieri S. The diagnosis and classification of undifferentiated connective tissue diseases. *J Autoimmun* 2014; **48–49**: 50–2.

3 Shiboski CH, Shiboski SC, Seror R, *et al.* 2016 American College of Rheumatology/European League Against Rheumatism Classification Criteria for Primary Sjögren’s Syndrome: A Consensus and Data-Driven Methodology Involving Three International Patient Cohorts. *Arthritis Rheumatol Hoboken NJ* 2017; **69**: 35–45.

4 Devreese KMJ, de Groot PG, de Laat B, *et al.* Guidance from the Scientific and Standardization Committee for lupus anticoagulant/antiphospholipid antibodies of the International Society on Thrombosis and Haemostasis. *J Thromb Haemost* 2020; **18**: 2828–39.

5 Amigo M-C, Goycochea-Robles MV, Espinosa-Cuervo G, *et al.* Development and initial validation of a damage index (DIAPS) in patients with thrombotic antiphospholipid syndrome (APS). *Lupus* 2015; **24**: 927–34.

6 Schulman S, Kearon C, the SUBCOMMITTEE ON CONTROL OF ANTICOAGULATION OF THE SCIENTIFIC AND STANDARDIZATION COMMITTEE OF THE INTERNATIONAL SOCIETY ON THROMBOSIS AND HAEMOSTASIS. Definition of major bleeding in clinical investigations of antihemostatic medicinal products in non-surgical patients: Definitions of major bleeding in clinical studies. *J Thromb Haemost* 2005; **3**: 692–4.

7 Kaatz S, Ahmad D, Spyropoulos AC, Schulman S, the Subcommittee on Control of Anticoagulation. Definition of clinically relevant non-major bleeding in studies of anticoagulants in atrial fibrillation and venous thromboembolic disease in non-surgical patients: communication from the SSC of the ISTH. *J Thromb Haemost* 2015; **13**: 2119–26.

**Supplementary Table S1. Distribution of cumulative thrombotic manifestations.**

| **Thrombotic manifestations** | **Patients**  **N = 116** | | **Events**  **N = 246** | |
| --- | --- | --- | --- | --- |
|  | N | % | N | % |
| **Venous** | 106 | 91.4 | 127 | 51.6 |
| Deep venous thrombosis | 53 | 45.7 | 62 | 25.2 |
| Lower limb | 47 | 40.5 | 56 | 22.8 |
| Upper limb | 3 | 2.6 | 3 | 1.2 |
| Other ^a^ | 3 | 2.6 | 3 | 1.2 |
| Pulmonary embolism | 23 | 19.8 | 24 | 9.8 |
| Central venous sinus thrombosis | 5 | 4.3 | 6 | 2.4 |
| Superficial venous thrombosis | 18 | 15.5 | 28 | 11.4 |
| Retinal vein thrombosis | 2 | 1.7 | 2 | 0.8 |
| Intra-abdominal venous thrombosis | 5 | 4.3 | 5 | 2.0 |
| Renal vein thrombosis | 0 | 0.0 | 0 | 0.0 |
| Portal vein thrombosis | 1 | 0.9 | 1 | 0.4 |
| Liver vein thrombosis | 1 | 0.9 | 1 | 0.4 |
| Spleen vein thrombosis | 2 | 1.7 | 2 | 0.8 |
| Mesenteric vein thrombosis | 1 | 0.9 | 1 | 0.4 |
| **Arterial** | 89 | 76.7 | 112 | 45.5 |
| Stroke | 40 | 34.5 | 50 | 20.3 |
| Transient ischemic attack | 14 | 12.1 | 21 | 8.5 |
| Myocardial infarction | 8 | 6.9 | 11 | 4.5 |
| Retinal artery thrombosis | 3 | 2.6 | 5 | 2.0 |
| Intra-abdominal arterial thrombosis | 14 | 12.1 | 14 | 5.7 |
| Aortic thrombosis | 5 | 4.3 | 5 | 2.0 |
| Renal artery thrombosis | 3 | 2.6 | 3 | 1.2 |
| Liver artery thrombosis | 1 | 0.9 | 1 | 0.4 |
| Spleen artery thrombosis | 2 | 1.7 | 2 | 0.8 |
| Mesenteric artery thrombosis | 3 | 2.6 | 3 | 1.2 |
| Lower limb ischaemia | 6 | 5.2 | 7 | 2.8 |
| Upper limb ischaemia | 4 | 3.4 | 4 | 1.6 |
| **Other** | 2 | 1.7 | 2 | 0.8 |
| Intracardiac thrombosis | 2 | 1.7 | 2 | 0.8 |
| **Microvascular thrombosis** | 4 | 3.4 | 5 | 2.0 |
| Renal microangiopathy ^b^ | 3 | 2.6 | 3 | 1.2 |
| Skin thrombosis ^c^ | 1 | 0.9 | 2 | 0.8 |

^a^ Other include internal jugular vein thrombosis (n=3). ^b^ Only one patient has biopsy-proven microvascular involvement. The remaining two patients were diagnosed based on clinical and laboratory criteria, including acute kidney injury and/or worsening of previous renal function, haematuria and/or proteinuria without any other identifiable cause. ^c^ Diagnosed based on clinical criteria (purpura retiform with skin ulcers, n=1) without any other identifiable cause.

**Supplementary Table S2. Comparison of the prevalence of aPL profile positivity and medical disorders at diagnosis and at the end of the study in all patients.**

|  | **At diagnosis** | | | **End of study** | | | **p-value** | | |
| --- | --- | --- | --- | --- | --- | --- | --- | --- | --- |
|  | **All** | **aPL-*negativization*** | | **All** | **aPL-*negativization*** | | **All vs all** | **No vs No** | **Yes vs Yes** |
|  |  | **No** | **Yes** |  | **No** | **Yes** |  |  |  |
| High blood pressure | 30 (25.9) | 23 (29.1) | 7 (18.9) | 58 (50.0) | 42 (53.2) | 16 (43.2) | **<0.001** | **0.002** | **0.024** |
| Hyperlipidaemia | 19 (16.4) | 12 (15.2) | 7 (18.9) | 58 (50.0) | 42 (53.2) | 16 (43.2) | **<0.001** | **<0.001** | **0.024** |
| Obesity | 25 (21.6) | 20 (25.3) | 5 (13.5) | 30 (25.9) | 24 (30.4) | 6 (16.2) | 0.440 | 0.478 | 0.744 |
| Diabetes | 4 (3.5) | 4 (5.1) | 0 | 8 (6.9) | 7 (8.9) | 1 (2.7) | 0.236 | 0.348 | 0.314 |
| Chronic kidney disease | 4 (3.5) | 3 (3.8) | 1 (2.7) | 12 (10.3) | 9 (11.4) | 3 (8.1) | **0.038** | 0.072 | 0.304 |
| Lupus anticoagulant | 75 (64.7) | 57 (72.2) | 18 (48.7) | 55 (47.4) | 52 (65.8) | 3 (8.1) | **0.008** | 0.390 | **<0.001** |
| aCL (IgM/IgG) | 97 (83·6) | 67 (84·8) | 30 (81.1) | 66 (56.9) | 66 (83.5) | 0 | **<0.001** | 1.000 | **<0.001** |
| aβ2GPI (IgM/IgG) | 75 (64.7) | 58 (73.4) | 17 (45.9) | 65 (56.0) | 65 (82.3) | 0 | 0.180 | 0.180 | **<0.001** |
| Triple positivity | 47 (40.5) | 43 (54.4) | 4 (10.8) | 43 (37.1) | 43 (54.4) | 0 | 0.590 | 1.000 | **0.040** |

Data are shown as number (%). aβ2GPI, anti-β2-glycoprotein I, aCL, anticardiolipin. Comparison was made using Test of proportions. Significant results are highlighted in bold.

**Supplementary Table S3. Frequency of aPL testing and testing consistency according to aPL-negativization status and recurrent thrombosis.**

|  |  | **aPL-negativization** | | | **Recurrent thrombosis** | | |
| --- | --- | --- | --- | --- | --- | --- | --- |
|  | **Total** | **Yes**  **N = 37** | **No**  **N = 79** | **p-value** | **Yes**  **N = 45** | **No**  **N = 71** | **p-value** |
| **Total number of aPL measurements** | 8.5 (6 - 12) | 9 (7 - 13) | 8 (6 - 11) | **0.048** | 8 (5 - 12) | 9 (7 - 13) | 0.273 |
| **aPL testing per year follow-up** | 1.3 (0.8 - 2.1) | 1.4 (0.9 - 2.0) | 1.2 (0.7 - 2.4) | 0.536 | 1.0 (1.2 - 0.9) | 1.5 (0.9 - 2.3) | **0.003** |
| **aPL testing consistency ^a^** | 77 (66.4) | 21 (56.8) | 56 (70.9) | 0.133 | 28 (62.2) | 49 (69.0) | 0.451 |

Data are shown as median (interquartile range). aPL, antiphospholipid antibodies. ^a^ Consistent in-house longitudinal testing using the same assay kits (see methods).

**Supplementary Table S4. Descriptive analysis on the aPL testing practices during the study period.**

|  |  |  | **Same aPL assays** | | **Total** |
| --- | --- | --- | --- | --- | --- |
|  |  |  | **No** | **Yes** |  |
| **Same laboratory** | **No** | **All patients** | **11 (100·0 \| 28**·**2)** | **0 (0**·**0 \| 0**·**0)** | **11 (100**·**0 \| 9**·**5)** |
|  |  | Become aPL-negative | 9 (100·0 \| 56·3) | 0 (0·0 \| 0·0) | 9 (100·0 \| 24·3) |
|  |  | Remain aPL-positive | 2 (100·0 \| 8·7) | 0 (0·0 \| 0·0) | 1 (100·0 \| 2·5) |
|  | **Yes** | **All patients** | **28 (26**·**7 \| 71**·**8)** | **77 (73**·**3 \| 100**·**0)** | **105 (100**·**0 \| 90**·**5)** |
|  |  | Become aPL-negative | 7 (25·0 \| 43·8) | 21 (75·0 \| 100·0) | 28 (100·0 \| 75·7) |
|  |  | Remain aPL-positive | 21 (27·3 \| 81·3) | 56 (76·7 \| 100·0) | 77 (100·0 \| 97·5) |
| **Total** | | **All patients** | **39 (33**·**6 \| 100**·**0)** | **77 (66**·**4 \| 100**·**0)** | **116 (100**·**0 \| 100**·**0)** |
|  |  | Become aPL-negative | 16 (43·2 \| 100·0) | 21 (56·8 \| 100·0) | 37 (100·0 \| 100·0) |
|  |  | Remain aPL-positive | 23 (29·1 \| 100·0) | 56 (70·9 \| 100·0) | 79 (100·0 \| 100·0) |

Data are shown as number (%). aPL, antiphospholipid antibodies.

**Supplementary Table S5. Comparison of demographic and clinical characteristics between patients with and without aPL-*negativization* and recurrent thrombosis.**

|  | **Total**  **N = 116** | **aPL-negativization** | | | **Recurrent thrombosis** | | |
| --- | --- | --- | --- | --- | --- | --- | --- |
|  |  | **Yes**  **N = 37** | **No**  **N = 79** | **p-value** | **Yes**  **N = 45** | **No**  **N = 71** | **p-value** |
| **Clinical characteristics** |  |  |  |  |  |  |  |
| Male sex | 25 (21.6) | 9 (24.3) | 16 (20.3) | 0.634 | 11 (24.4) | 14 (19.7) | 0.644 |
| Current age (years) | 52 (42.5 – 63) | 52 (40 – 60) | 52 (43 – 63) | 0.694 | 58 (46 – 66) | 51 (40 – 59) | **0.034** |
| Age at APS onset (years) | 38 (25 – 49.5) | 37 (26 – 47) | 39 (24 – 52) | 0.455 | 41 (28 – 51) | 38 (24 – 48) | 0.491 |
| Time of follow-up (months) | 99 (46.5 – 186.5) | 88 (62 – 149) | 111 (38 – 192) | 0.681 | 121 (65 – 191) | 88 (39 – 161) | **0.047** |
| **APS characterization** |  |  |  |  |  |  |  |
| Primary APS | 72 (72.1) | 26 (70.3) | 46 (58.2) | 0.213 | 26 (57.8) | 46 (64.8) | 0.448 |
| Secondary APS | 44 (37.9) | 11 (29.7) | 33 (41.8) |  | 19 (42.2) | 25 (35.2) |  |
| Type of first thrombotic event |  |  |  |  |  |  |  |
| Arterial | 57 (49.1) | 17 (44.9) | 40 (50.6) | 0.693 | 27 (60.0) | 30 (42.3) | 0.062 |
| Venous | 58 (50.0) | 20 (54.1) | 38 (48.1) | 0.550 | 18 (40.0) | 40 (56.3) | 0.127 |
| Microvascular ^a^ | 1 (0.9) | 0 | 1 (1.3) | - | 0 | 1 (1.4) |  |
| Obstetric morbidity | 22/73 (30.1) | 6/24 (25.0) | 16 (32.7) | 0.594 | 12/27 (44.4) | 10/46 (21.7) | 0.064 |
| **aPL profile** |  |  |  |  |  |  |  |
| At diagnosis |  |  |  |  |  |  |  |
| Lupus anticoagulant | 75 (64.7) | 18 (48.7) | 57 (72.2) | **0.021** | 33 (73.3) | 42 (59.2) | 0.120 |
| Anticardiolipin (IgM/IgG) | 97 (83.6) | 30 (81.1) | 67 (84.8) | 0.613 | 40 (88.9) | 57 (80.3) | 0.222 |
| aβ2GPI (IgM/IgG) | 75 (64.7) | 17 (45.9) | 58 (73.4) | **0.006** | 30 (66.7) | 45 (63.4) | 0.718 |
| Triple positive | 47 (40.5) | 4 (10.8) | 43 (54.4) | **<0.001** | 23 (51.1) | 24 (33.8) | 0.064 |
| At end of study |  |  |  |  |  |  |  |
| Lupus anticoagulant | 55 (47.4) | 3 (8.1) | 52 (65.8) | **<0.001** | 24 (53.3) | 31 (43.7) | 0.309 |
| Anticardiolipin (IgM/IgG) | 66 (56.9) | 0 | 66 (83.5) | **<0.001** | 32 (71.1) | 34 (47.9) | **0.014** |
| aβ2GPI (IgM/IgG) | 65 (56.0) | 0 | 65 (82.3) | **<0.001** | 30 (66.7) | 35 (49.3) | 0.066 |
| Triple positive | 43 (37.1) | 0 | 43 (54.4) | **<0.001** | 23 (51.1) | 20 (28.2) | **0.013** |
| aPL-negativization | 37 (31.9) | - | - | - | 11 (24.4) | 26 (36.6) | 0.221 |
| **Medical disorders** |  |  |  |  |  |  |  |
| At diagnosis |  |  |  |  |  |  |  |
| High blood pressure | 30 (25.9) | 7 (18.9) | 23 (29.1) | 0.266 | 11 (24.4) | 19 (26.8) | 0.831 |
| Hyperlipidaemia | 19 (16.4) | 7 (18.9) | 12 (15.2) | 0.601 | 9 (20.0) | 10 (14.1) | 0.446 |
| Obesity | 25 (21.6) | 5 (13.5) | 20 (25.3) | 0.225 | 9 (20.0) | 16 (22.5) | 0.820 |
| Diabetes | 4 (3.5) | 0 | 4 (5.1) | - | 1 (2.2) | 3 (4.2) | 1.000 |
| Chronic kidney disease | 4 (3.5) | 1 (2.7) | 3 (3.8) | 1.000 | 1 (2.2) | 3 (4.2) | 1.000 |
| At end of study |  |  |  |  |  |  |  |
| High blood pressure | 58 (50.0) | 16 (43.2) | 42 (53.2) | 0.426 | 21 (46.7) | 37 (52.1) | 0.568 |
| Hyperlipidaemia | 58 (50.0) | 16 (43.2) | 42 (53.2) | 0.426 | 23 (55.6) | 33 (46.5) | 0.341 |
| Obesity | 30 (25.9) | 6 (16.2) | 24 (30.4) | 0.117 | 10 (22.2) | 20 (28.2) | 0.521 |
| Diabetes | 8 (6.9) | 1 (2.7) | 7 (8.9) | 0.433 | 2 (4.4) | 6 (8.5) | 0.481 |
| Chronic kidney disease | 12 (10.3) | 3 (8.1) | 9 (11.4) | 0.749 | 4 (8.9) | 8 (11.3) | 0.764 |
| Thrombophilia ^b^ | 6/59 (10.2) | 3/20 (15.0) | 3/39 (7.7) | 0.398 | 4/22 (18.2) | 2/37 (5.4) | 0.183 |
| Smoking | 57 (49.1) | 17 (45.9) | 40 (50.6) | 0.693 | 25 (55.6) | 32 (45.1) | 0.271 |
| **Treatment (ever-present)** |  |  |  |  |  |  |  |
| Anticoagulation | 108 (93.1) | 32 (86.5) | 76 (96.2) | 0.054 | 44 (97.8) | 64 (90.1) | 0.114 |
| VKA | 104 (89.7) | 31 (83.8) | 73 (92.4) | 0.155 | 44 (97.8) | 60 (84.5) | **0.022** |
| DOAC ^c^ | 23 (19.8) | 5 (13.5) | 18 (22.8) | 0.321 | 8 (17.8) | 15 (21.1) | 0.812 |
| Antiplatelets ^d^ | 67 (57.8) | 23 (62.2) | 44 (55.7) | 0.511 | 31 (68.9) | 36 (50.7) | 0.053 |
| Statin | 64 (55.2) | 18 (48.7) | 46 (58.2) | 0.423 | 32 (71.1) | 32 (45.1) | **0.006** |
| Hydroxychloroquine | 50 (43.1) | 16 (43.2) | 34 (43.0) | 1.000 | 22 (48.9) | 28 (39.4) | 0.316 |
| Steroids | 37 (31.9) | 9 (24.3) | 28 (35.4) | 0.288 | 19 (42.2) | 18 (25.4) | 0.068 |
| DMARDs | 25 (21.6) | 9 (24.3) | 16 (20.3) | 0.634 | 11 (24.4) | 14 (19.7) | 0.644 |
| Azathioprine | 19 (16.4) | 6 (16.2) | 13 (16.5) | 1.000 | 8 (17.8) | 11 (15.5) | 0.800 |
| Methotrexate | 6 (5.2) | 1 (2.7) | 5 (6.3) | 0.663 | 3 (6.7) | 3 (4.2) | 0.676 |
| Mycophenolate mofetil | 7 (6.0) | 2 (5.4) | 5 (6.3) | 1.000 | 3 (6.7) | 4 (5.6) | 1.000 |
| Other ^e^ | 5 (4.3) | 3 (9.1) | 2 (2.5) | 0.325 | 3 (6.7) | 2 (2.8) | 0.375 |
| Biologics | 5 (4.3) | 2 (5.4) | 3 (3.8) | 0.653 | 1 (2.2) | 4 (5.6) | 0.647 |
| Rituximab | 4 (3.5) | 2 (5.4) | 2 (2.5) | 0.591 | 1 (2.2) | 3 (4.2) | 1.000 |
| Other ^f^ | 1 (0.9) | 0 | 1 (1.3) | - | 0 | 1 (1.4) | - |
| **Outcomes** |  |  |  |  |  |  |  |
| Number thrombotic events | 2 (1 – 3) | 2 (1 – 2) | 2 (1 – 3) | 0.158 | 3 (2 – 4) | 1 (1 – 2) | **<0.001** |
| DIAPS ≥1 | 89 (76.7) | 29 (78.4) | 60 (75.9) | 0.773 | 40 (88.9) | 49 (69.0) | **0.014** |
| Early damage ^g^ | 48 (41.4) | 18 (48.7) | 30 (38.0) | 0.277 | 17 (37.8) | 31 (43.7) | 0.567 |
| DIAPS initial | 0 (0 – 1)  0.5 ± 0.6 | 0 (0 – 1)  0.5 ± 0.5 | 0 (0 – 1)  0.4 – 0.6 | 0.447 | 0 (0 – 1)  0.4 ± 0.5 | 0 (0 – 1)  0.5 ± 0.6 | 0.371 |
| DIAPS ≥3 | 37 (31.9) | 12 (32.4) | 25 (31.7) | 1.000 | 24 (53.3) | 13 (18.3) | **<0.001** |
| DIAPS final | 2 (1 – 3)  1.8 ± 1.5 | 1 (1 – 3)  1.7 ± 1.4 | 2 (1 – 3)  1.9 ± 1.6 | 0.796 | 3 (1 – 3)  2.4 ± 1.6 | 1 (0 – 2)  1.4 ± 1.4 | **<0.001** |

Data are shown as number (%) and median (interquartile range) when appropriate. DIAPS is also reported as mean ± standard deviation for comparison with similar studies. The denominator is provided if it differs from the group total. Significant results are highlighted in bold. See **Supplementary Data 1** for further detail on medical disorders’ definitions. ^a^ These include renal thrombotic microangiopathy and microvascular skin thrombosis. ^b^ Inherited thrombophilia included the following: protein S deficiency (n=4/76, 5.3%), factor V Leiden (n=3/66, 4.5%), 20210a prothrombin mutation (n=2/64, 3.1%), and protein C deficiency (n=2/78, 2.6%). ^c^ DOAC included the following: Rivaroxaban (n=15), apixaban (n=7), and dabigatran (n=1). ^d^ Antiplatelets included the following: low-dose aspirin (n=65), and clopidogrel (n=2). ^e^ Other DMARDs included the following: cyclophosphamide (n=5), intravenous immunoglobulin (n=2), and ciclosporin (n=2). ^f^ Other biologics included the following: secukinumab (n=1). ^g^ Early damage refers to damage acquired during the first six months after disease onset. ^h^ Classified according to International Society on Thrombosis and Haemostasis definitions ^6,7^.

aβ2GPI, anti-β2-glycoprotein I; aPL, antiphospholipid antibodies; APS, antiphospholipid syndrome; DIAPS, damage index for antiphospholipid syndrome; DMARDs, disease modifying anti-rheumatic drugs; DOAC, direct oral anticoagulant; VKA, vitamin K antagonist; y, years.

**Supplementary Table S6. Stratified Cox regression analysis by aPL specificity and isotype.**

|  | **aPL-negativization** | | **Recurrent thrombosis** | |
| --- | --- | --- | --- | --- |
| **At diagnosis** | **HR (95% CI)** | **p-value** | **HR (95% CI)** | **p-value** |
| aCL and/or aβ2GPI (IgM) | 1.81 (0.94 – 3.50) | 0.076 | 0.64 (0.32 – 1.27) | 0.205 |
| Double-positive for aCL and aβ2GPI (IgM) | 0.27 (0.08 – 0.88) | **0.031** | 0.96 (0.47 – 1.94) | 0.903 |
| Single-positive for aCL (IgG/IgM) | 3.39 (1.77 – 6.49) | **<0.001** | 0.88 (0.46 – 1.68) | 0.694 |
| Single-positive for aCL (IgM) | 2.18 (1.03 – 4.64) | **0.042** | 0.80 (0.34 – 1.92) | 0.619 |
| Single-positive for aβ2GPI (IgG/IgM) | 2.74 (1.19 – 6.27) | **0.017** | 0.57 (0.18 – 1.87) | 0.360 |
| Single-positive for aβ2GPI (IgM) | 3.13 (1.01 – 8.92) | **0.032** | 0.37 (0.05 – 2.71) | 0.330 |
| **End of study** |  |  |  |  |
| aCL and/or aβ2GPI (IgM) | - | - | 1.16 (0.54 – 2.49) | 0.710 |
| Double-positive for aCL and aβ2GPI (IgM) | - | - | 1.52 (0.60 – 3.90) | 0.379 |
| Single-positive for aCL (IgG/IgM) | - | - | 1.08 (0.39 – 3.04) | 0.882 |
| Single-positive for aCL (IgM) | - | - | 0.82 (0.17 – 2.96) | 0.644 |
| Single-positive for aβ2GPI (IgG/IgM) | - | - | 0.37 (0.09 – 1.52) | 0.168 |
| Single-positive for aβ2GPI (IgM) | - | - | 1.09 (0.15 – 7.98) | 0.930 |

Data are shown as univariate hazard ratio (HR) and 95% confidence interval (CI). aβ2GPI, anti-β2 glycoprotein I; aCL, anticardiolipin.

**Supplementary Table S7. Treatment practices regarding the maintenance, discontinuation or initiation of anticoagulation.**

|  | **Total**  **N= 9** | **aPL-*negativization*** | |
| --- | --- | --- | --- |
|  |  | **Yes**  **N = 6** | **No**  **N = 3** |
| Male sex | 1 (11.1) | 1(16.7) | 0 |
| Total follow-up time (months) | 99 (46 – 180) | 71 (39 – 157) | 99 (39 – 157) |
| Follow-up time after aPL-*negativization* (months) | 34 (14 – 89) | 34 (14 – 89) | - |
| Type of first thrombosis (venous) | 8 (88.9) | 5 (83.3) | 3 (100.0) |
| Primary APS | 5 (55.6) | 4 (66.7) | 1 (33.3) |
| aPL profile (at diagnosis) |  |  |  |
| Lupus anticoagulant | 4 (44.4) | 2 (33.3) | 2 (66.7) |
| Anticardiolipin | 8 (88.9) | 6 (100.0) | 2 (66.7) |
| Anti-β2-glycoprotein I | 4 (44.4) | 2 (33.3) | 2 (66.7) |
| Triple | 1 (11.1) | 0 | 1 (33.3) |
| aPL profile (at end of study) |  |  |  |
| Lupus anticoagulant | 2 (22.2) | 0 | 2 (66.7) |
| Anticardiolipin | 2 (22.2) | 0 | 2 (66.7) |
| Anti-β2-glycoprotein I | 2 (22.2) | 0 | 2 (66.7) |
| Triple | 1 (11.1) | 0 | 1 (33.3) |
| Treatment (ever-present) |  |  |  |
| Anticoagulation | 9 (100.0) | 6 (100.0) | 3 (100.0) |
| Vitamin K antagonist | 6 (66.7) | 5 (83.3) | 1 (33.3) |
| Direct oral anticoagulant | 2 (22.2) | 1 (16.7) | 1 (33.3) |
| Low molecular weight heparin | 1 (11.1) | 0 | 1 (33.3) |
| Antiplatelets | 7 (77.8) | 4 (66.7) | 3 (100.0) |
| Treatment (current) |  |  |  |
| Anticoagulation | 0 | 0 | 0 |
| Vitamin K antagonist | 0 | 0 | 0 |
| Direct oral anticoagulant | 0 | 0 | 0 |
| Low molecular weight heparin | 0 | 0 | 0 |
| Antiplatelets | 7 (77.8) | 4 (66.7) | 3 (100.0) |
| Recurrent thrombosis | 0 | 0 | 0 |

Data are shown as number (%) and median (interquartile range) when appropriate. aPL, antiphospholipid antibodies; APS, antiphospholipid syndrome.

**Supplementary Table S8. Characterization of recurrent thrombosis in patients who become aPL-negative.**

| **ID** | **Sex** | **Type \| Condition** | **Age (y)**  **Current \| Onset** | **Diagnosis (date)** | **Follow-up (mo)** | **First thrombosis** | **aPL-profile** | **aPL-negativization dates** | | **Thrombotic recurrence** | | | | **Treatment regimen ^b^** | | | **Treatment intensity ^b^** | |
| --- | --- | --- | --- | --- | --- | --- | --- | --- | --- | --- | --- | --- | --- | --- | --- | --- | --- | --- |
|  |  |  |  |  |  |  |  | **1^st^** | **2^nd^** | **Number** | **Before ^a^** | **After ^a^** | **Specification (date)** | **Ever** | **Near recurrence** | **Last ^c^** | **Last ^d^** | **Near recurrence** ^e^ |
| 2 | M | PAPS \| - | 69 \| 54 | 07/2009 | 95 | Arterial | LA; aCL IgM | 09/2018 | 01/2021 | 1 | Yes | No | Myocardial infarction (12/2015) | W, AAS | AAS | W | 2-3 | - |
| 5 | M | PAPS \| - | 66 \| 59 | 02/2017 | 84 | Arterial | LA; aCL IgM | 09/2019 | 04/2022 | 1 | No | Yes | Anterior ischaemic optic neuropathy (08/2023) | W | W | W | 2.5-3.5 | - |
| 61 | F | PAPS \| - | 58 \| 52 | 10/2015 | 61 | Arterial | aCL IgG | 01/2020 | 11/2021 | 1 | Yes | No | Lower limb DVT (01/2018) | AAS | AAS | AAS | - | - |
| 83 | F | PAPS \| - | 43 \| 37 | 10/2018 | 65 | Arterial | aCL IgG; aβ2GPI IgG | 08/2019 | 02/2023 | 2 | Yes | Yes | TIA (04/2019); TIA (10/2023) | W, AAS | AAS \| W | W | 2-3 | 3.1 |
| 87 | F | PAPS \| - | 68 \| 63 | 09/2020 | 39 | Arterial | aCL IgM; aβ2GPI IgM | 06/2023 | 06/2024 | 2 | Yes | No | Stroke (04/2021); Stroke (06/2021) | W, AAS | AAS \| W | W | 2-3 | - \| - |
| 125 | F | PAPS \| - | 80 \| 26 | 01/1993 | 149 | Arterial | LA; aCL IgM | 12/2017 | 01/2019 | 2 | Yes | Yes | Myocardial infarction (12/2017); Lower limb DVT (08/2019) | W, LMWH, AAS | AAS \| AAS | W, AAS | 2-3 | - \| - |
| 140 | F | PAPS \| - | 58 \| 47 | 09/2013 | 66 | Venous | aCL IgG | 01/2020 | 01/2021 | 1 | Yes | No | Lower limb SVT (08/2015) | W | W | W | 2-3 | - |
| 176 | F | PAPS \| - | 60 \| 48 | 01/2013 | 35 | Arterial | aCL IgG | 03/2020 | 05/2021 | 4 | Yes | No | TIA (09/2017); Stroke (09/2018); TIA (01/2019); TIA (01/2020) | W, AAS | AAS \| AAS \| AAS \| AAS | W, AAS | 2-3 | - |
| 225 | F | SAPS \| SLE | 40 \| 22 | 01/2006 | 65 | Venous | LA; aβ2GPI IgG | 11/2020 | 05/2022 | 1 | Yes | No | Lower limb DVT (09/2013) | W | W | W | 2-3 | 1.2 |
| 246 | F | SAPS \| UCTD | 52 \| 46 | 06/2018 | 78 | Arterial | LA; aCL IgG; aβ2GPI IgG | 06/2021 | 07/2023 | 1 | Yes | No | Stroke (10/2020) | W | W | W | 2-3 | 1.6 |
| 248 | F | SAPS \| SLE | 28 \| 18 | 01/2014 | 84 | Venous | LA; aβ2GPI IgG | 12/2021 | 01/0124 | 1 | Yes | No | CAPS with bilateral kidney infarct needing dialysis, right toe ischaemia, and retinal artery thrombosis (09/2021) | W, RVX | RVX | W | 2-3 | Standard |

Data are shown when available. Each row represents a distinct patient. ^a^ In respect to the time of aPL-negativization. ^b^ Whenever there was more than one recurrent thrombosis, data are separated by a “ | ” and presented chronologically. ^c^ Latest treatment regimen. ^d^ Latest INR target value / aimed anticoagulation intensity. ^e^ Standard included rivaroxaban 20mg id.

AAS, acetylsalicylic acid; aβ2GPI, anti-β2-glycoprotein I; aCL, anticardiolipin; CAPS, catastrophic antiphospholipid syndrome; DVT, deep vein thrombosis; F, female; LA, lupus anticoagulant; LMWH, low molecular weight heparin; M, male; mo, months; PAPS, primary antiphospholipid syndrome; RVX, rivaroxaban; SAPS, secondary antiphospholipid syndrome; SLE, systemic lupus erythematosus; SVT, superficial vein thrombosis; TIA, transient ischemic attack; UCTD, undifferentiated connective tissue disease; W, warfarin; y, years.

**Supplementary Table S9.** **Characterization of recurrent thrombosis in patients who remained aPL-positive.**

| **ID** | **Sex** | **Type \| Condition** | **Age (y) \| Onset (y)** | **Diagnosis (y)** | **Follow-up (mo)** | **First thrombotic event** | **aPL-profile** | **Thrombotic recurrence** | | **Treatment regimen ^a^** | | | **Treatment intensity ^a^** | |
| --- | --- | --- | --- | --- | --- | --- | --- | --- | --- | --- | --- | --- | --- | --- |
|  |  |  |  |  |  |  |  | **Number** | **Specification (date)** | **Ever** | **Near recurrence** | **Last ^b^** | **Last ^c,d^** | **Near recurrence ^d^** |
| 36 | F | PAPS \| - | 39 \| 9 | 01/1992 | 165 | Arterial | LA; aCL IgG and IgM; aβ2GPI IgG | 1 | Pulmonary embolism (03/2011) | W, AAS | W | W | 2 - 3 | 1.3 |
| 43 | F | PAPS \| - | 64 \| 46 | 01/2006 | 206 | Arterial | LA; aCL IgG and IgM; aβ2GPI IgG and IgM | 2 | Right toe ischaemia (03/2019); Aortic and renal artery thrombosis (03/2021) | W, AAS | W \| W | W, AAS | 2 - 3 | 1.3 \| 3.3 |
| 56 | F | PAPS \| - | 43 \| 32 | 04/2013 | 56 | Arterial | aCL IgG | 2 | Myocardial infarction (01/2017); stroke (01/2019) | W, AAS | AAS \| AAS | W | 2 - 3 | - \| - |
| 65 | M | PAPS \| - | 55 \| 49 | 05/2018 | 67 | Arterial | LA; aCL IgM; aβ2GPI IgM | 1 | Pulmonary embolism and aortic thrombosis (03/2019) | W, LMWH | AAS | W | 2 - 3 | - |
| 68 | F | PAPS \| - | 45 \| 29 | 01/2008 | 191 | Arterial | LA; aCL IgG; aβ2GPI IgG | 2 | Intracardiac thrombus (01/2014); CSVT (01/2019) | W, AAS | W \| W | W | 2.5 - 3.5 | 1.0 \| - |
| 69 | F | PAPS \| - | 45 \| 22 | 01/2001 | 121 | Venous | LA; aCL IgG and IgM; aβ2GPI IgG and IgM | 2 | SVT lower limb (12/2016); Lower limb DVT (10/2020) | W, AAS | AAS \| AAS | W | 2 - 3 | - \| - |
| 71 | M | PAPS \| - | 80 \| 64 | 01/2008 | 165 | Arterial | LA; aCL IgG; aβ2GPI IgG | 1 | Upper limb SVT (11/2010) | W, RVX, AAS | W | RVX, AAS | Standard | 1.1 |
| 72 | F | PAPS \| - | 52 \| 35 | 11/2007 | 204 | Arterial | LA; aCL IgG; aβ2GPI IgG | 1 | Lower limb DVT (09/2008) | W, AAS | AAS | W | 2 - 3 | - |
| 73 | M | SAPS \| SSjo | 70 \| 52 | 01/2007 | 128 | Venous | LA; aCL IgG and IgM; aβ2GPI IgG | 1 | Lower limb SVT (09/2020) | W | W | W | 2 - 3 | - |
| 74 | M | PAPS \| - | 52 \| 32 | 01/2004 | 27 | Venous | LA; aCL IgG; aβ2GPI IgG | 1 | Right toe ischemia (03/2019) | W, RVX | RVX | W | 2 - 3 | Standard |
| 78 | F | PAPS \| - | 50 \| 28 | 01/2002 | 104 | Venous | aCL IgM; aβ2GPI IgM | 3 | Tia (01/2012); Lower limb DVT (10/2015); Lower limb SVT (04/2022) | W, AAS | AAS \| AAS \| W | W | 2.5 - 3.5 | - \| - \| - |
| 81 | M | PAPS \| - | 48 \| 43 | 09/2018 | 46 | Arterial | aβ2GPI IgM | 1 | Retinal vein thrombosis (09/2021) | W, AAS | AAS | W, AAS | 2 - 3 | . |
| 85 | F | PAPS \| - | 58 \| 50 | 12/2015 | 32 | Arterial | LA; aCL IgG; aβ2GPI IgG | 2 | Stroke (11/2019); stroke (02/2021) | W, Clopi | Clopi \| Clopi | W | 3 - 4 | - \| - |
| 97 | F | PAPS \| - | 24 \| 21 | 05/2021 | 38 | Venous | LA; aCL IgG; aβ2GPI IgG | 1 | CSVT (01/2022) | W, Apixa | Apixa | W | 2 - 3 | Standard |
| 120 | F | PAPS \| - | 71 \| 56 | 01/2010 | 131 | Arterial | aCL IgM; aβ2GPI IgM | 1 | Upper limb DVT (04/2024) | W, AAS | AAS | W, AAS | 2 - 3 | - |
| 127 | F | PAPS \| - | 34 \| 22 | 09/2013 | 116 | Arterial | LA; aCL IgG; aβ2GPI IgG | 2 | Aortic thrombosis (01/2015); Lower limb arterial thrombosis (11/2019) | W, LMWH, AAS, Clopi | AAS \| W, AAS | W, AAS | 2 - 3 | - \| 1.5 |
| 144 | M | PAPS \| - | 59 \| 53 | 01/2018 | 61 | Arterial | LA; aCL IgG; aβ2GPI IgG | 1 | Aortic thrombosis (11/2019) | W, AAS | AAS | W, AAS | 3 - 4 | - |
| 159 | F | PAPS \| - | 60 \| 37 | 01/2001 | 255 | Venous | LA; aCL IgG; aβ2GPI IgG | 2 | Lower limb DVT (01/2018); lower limb arterial thrombosis (04/2024) | W, AAS | W \| W, AAS | W, AAS | 3 - 4 | - \| 2.7 |
| 191 | F | SAPS \|SLE | 63 \| 42 | 01/2003 | 157 | Venous | LA; aCL IgG and IgM; aβ2GPI IgG | 1 | Stroke (12/2010) | W | W | W | 2 - 3 | - |
| 194 | F | SAPS \| MCTD | 68 \| 51 | 01/2007 | 204 | Venous | aCL IgM | 2 | Pulmonary embolism (11/2013); lower limb DVT (05/2016) | W, AAS | W \| W | W | 2 - 3 | - \| - |
| 195 | F | SAPS \|SLE | 41 \| 25 | 01/2008 | 203 | Arterial | LA; aCL IgG; aβ2GPI IgG | 1 | TIA (05/2023) | W, LMWH, AAS | W | W, AAS | 3 - 4 | 8.2 |
| 197 | F | SAPS \|SLE | 76 \| 71 | 01/2019 | 39 | Arterial | LA; aCL IgG; aβ2GPI IgG | 1 | Myocardial infarction (12/2023) | W, RVX, LMWH, AAS | W | W | 2 - 3 | 2.1 |
| 199 | F | SAPS \|SLE | 59 \| 51 | 03/2016 | 99 | Arterial | LA; aCL IgG; aβ2GPI IgG | 2 | TIA (01/2018); TIA (01/2019) | W, AAS | AAS \| W | W, AAS | 2 - 3 | - \| - |
| 220 | F | SAPS \|SLE | 46 \| 28 | 01/2006 | 183 | Venous | LA; aCL IgG and IgM; aβ2GPI IgG | 1 | Stroke (08/2016) | W, LMWH | W | W | 2 - 3 | 2.2 |
| 222 | F | SAPS \|SLE | 60 \| 32 | 01/1997 | 194 | Arterial | LA; aCL IgG; aβ2GPI IgG | 4 | Stroke (01/2010); stroke (01/2011); TIA (03/2013); stroke (10/2014) | W, AAS | AAS \| AAS \| AAS \| AAS | W | 2 - 3 | - |
| 227 | F | SAPS \|SLE | 57 \| 39 | 01/2006 | 190 | Venous | LA; aCL IgG and IgM; aβ2GPI IgG and IgM | 2 | Stroke (09/2012); lower limb SVT and microvascular skin thrombosis (12/2013) | W, RVX, AAS | AAS \| RVX | RVX, AAS | Standard | - \| Standard |
| 229 | F | SAPS \|SLE | 29 \| 17 | 09/2012 | 125 | Venous | LA; aCL IgG and IgM; aβ2GPI IgG and IgM | 2 | Lower limb SVT (12/2013); lower limb DVT (05/2014) | W | W \| W | W | 2 - 3 | - \| 2.4 |
| 236 | F | SAPS \|SLE | 55 \| 45 | 20/2014 | 115 | Arterial | LA; aCL IgG; aβ2GPI IgG | 1 | Lower limb SVT (09/2021) | W, Apixa, AAS | Apixa | W | 2 - 3 | Standard |
| 238 | F | SAPS \|SLE | 58 \| 39 | 01/2006 | 192 | Venous | LA; aCL IgG and IgM; aβ2GPI IgG and IgM | 1 | Jugular vein thrombosis (07/2017) | W, AAS | AAS | W | 2 - 3 | - |
| 249 | F | SAPS \|SLE | 87 \| 71 | 01/2009 | 166 | Venous | LA; aCL IgG and IgM; aβ2GPI IgG and IgM | 1 | Stroke (12/2010) | W | W | W | 2 - 3 | 1.9 |
| 251 | F | SAPS \|SLE | 49 \| 24 | 01/1999 | 287 | Venous | LA; aCL IgG and IgM; aβ2GPI IgG and IgM | 2 | Lower limb DVT (01/2017); CAPS with pulmonary embolism, Budd-Chiari syndrome and livedoid vasculopathy (01/2021) | W, LMWH, Dabiga, AAS | Dabiga \| W | W, AAS | 2.5 - 3.5 | Standard \| - |
| 267 | F | SAPS \|SLE | 71 \| 41 | 01/1994 | 277 | Arterial | LA; aCL IgM; aβ2GPI IgM | 1 | Lower limb arterial thrombosis (12/2004) | W | W | W | 2.5 - 3.5 | 1.3 |
| 271 | F | SAPS \|SLE | 48 \| 17 | 01/1993 | 323 | Venous | LA; aCL IgM | 1 | Stroke (01/2016) | W, LMWH, AAS | W | W | 2.5 - 3.5 | 1.3 |
| 275 | F | SAPS \|SLE | 74 \| 47 | 01/1997 | 275 | Arterial | LA; aCL IgM; aβ2GPI IgM | 1 | TIA (01/2000) | W, AAS | AAS | W | 2 - 3 | - |

Data are shown when available. Each row represents a distinct patient. ^a^ Whenever there was more than one recurrent thrombosis, data are separated by a “ | ” and presented chronologically. ^b^ Latest treatment regimen. ^c^ Latest INR target value / aimed anticoagulation intensity. ^d^ Standard included rivaroxaban 20mg id, apixaban 5mg bid, and dabigatran 300mg bid.

AAS, acetylsalicylic acid; aβ2GPI, anti-β2-glycoprotein I; aCL, anticardiolipin; Apixa; apixaban; CAPS, catastrophic antiphospholipid syndrome; Clopi, clopidogrel; CSVT, cerebral sinus venous thrombosis; Dabiga; dabigatran; DVT, deep vein thrombosis; F, female; LA, lupus anticoagulant; LMWH, low molecular weight heparin; M, male; MCTD, mixed connective tissue disease; mo, months; PAPS, primary antiphospholipid syndrome; RVX, rivaroxaban; SAPS, secondary antiphospholipid syndrome; SLE, systemic lupus erythematosus; SVT, superficial vein thrombosis; SSjo, Sjögren syndrome; TIA, transient ischemic attack; W, warfarin; y, years.

**Supplementary Table S10. Multivariate hazard ratio identifying predictors of recurrent thrombosis.**

|  | **Recurrent thrombosis** | |
| --- | --- | --- |
|  | **HR (95% CI)** | **p-value** |
| Age at APS onset (years) ^a^ | 1.02 (0.99 – 1.05) | 0.089 |
| Primary APS | 1.10 (0.57 – 2.13) | 0.774 |
| Arterial thrombosis | 1.83 (0.92 – 3.61) | 0.083 |
| aPL-negativization | 0.19 (0.06 – 0.63) | **0.006** |

Data are shown as multivariate hazard ratio (HR) and 95% confidence interval (CI). Statistically significant results are highlighted in bold. ^a^ Per year increase HR. aPL, antiphospholipid antibodies; APS, antiphospholipid syndrome.
